# Exploring LoRaWAN Traffic: In-Depth Analysis of IoT Network Communications

**DOI:** 10.3390/s23177333

**Published:** 2023-08-22

**Authors:** Ales Povalac, Jan Kral, Holger Arthaber, Ondrej Kolar, Marek Novak

**Affiliations:** 1 Faculty of Electrical Engineering and Communication, Brno University of Technology, Technicka 12, 61600 Brno, Czech Republic; jan.kral@vut.cz (J.K.); ondrej.kolar@vut.cz (O.K.); xnovak0m@vut.cz (M.N.); 2 Institute of Electrodynamics, Microwave and Circuit Engineering, TU Wien, Gusshausstrasse 25/354, 1040 Vienna, Austria; holger.arthaber@tuwien.ac.at

**Keywords:** IoT, LoRa, LoRaWAN, Class-B, dataset, network sniffer, traffic monitoring, time synchronization

## Abstract

In the past decade, Long-Range Wire-Area Network (LoRaWAN) has emerged as one of the most widely adopted Low Power Wide Area Network (LPWAN) standards. Significant efforts have been devoted to optimizing the operation of this network. However, research in this domain heavily relies on simulations and demands high-quality real-world traffic data. To address this need, we monitored and analyzed LoRaWAN traffic in four European cities, making the obtained data and post-processing scripts publicly available. For monitoring purposes, we developed an open-source sniffer capable of capturing all LoRaWAN communication within the EU868 band. Our analysis discovered significant issues in current LoRaWAN deployments, including violations of fundamental security principles, such as the use of default and exposed encryption keys, potential breaches of spectrum regulations including duty cycle violations, SyncWord issues, and misaligned Class-B beacons. This misalignment can render Class-B unusable, as the beacons cannot be validated. Furthermore, we enhanced Wireshark’s LoRaWAN protocol dissector to accurately decode recorded traffic. Additionally, we proposed the passive reception of Class-B beacons as an alternative timebase source for devices operating within LoRaWAN coverage under the assumption that the issue of misaligned beacons can be addressed or mitigated in the future. The identified issues and the published dataset can serve as valuable resources for researchers simulating real-world traffic and for the LoRaWAN Alliance to enhance the standard to facilitate more reliable Class-B communication.

## 1. Introduction

The Internet of Things (IoT) has revolutionized the way we interact with our environment, enabling a wide range of applications from smart cities to industrial automation. LPWANs have emerged as key technology for IoT, providing a balance between low power consumption and long-range communication.

LoRaWAN, a popular LPWAN technology, is based on the Long-Range (LoRa) physical layer and provides features such as adaptive data rates, bidirectional communication, and various device classes, making it suitable for different use cases. Given the limited Radio Frequency (RF) power of 25 mW, LoRaWAN facilitates a communication distance of up to 5 km in urban areas [1]. These diverse capabilities have led to widespread adoption across various industries, establishing it as a vital component in the growing IoT ecosystem [2,3].

The LoRa Physical (PHY) layer employs a unique modulation technique known as Chirp Spread Spectrum (CSS). CSS facilitates long-range communication and robustness against narrow-band interference by spreading the information signal over a wider bandwidth [4]. Above this, the LoRaWAN Medium Access Control (MAC) layer provides a standardized protocol for IoT devices [5].

LoRaWAN features three distinct device classes—A, B, and C—addressing different application requirements and power constraints [5]. Class-A devices offer the highest energy efficiency, suitable for applications with infrequent communication needs, with brief receive windows after each transmission. Class-B devices provide predictable downlink communication latency by synchronizing with network beacons and enabling scheduled receive slots, maintaining moderate power consumption. Class-C devices prioritize downlink latency over power efficiency, offering continuous receive windows for near real-time communication. End devices use a random access transmission method (ALOHA), which allows them communication without the need for pairing with a specific gateway.

Given the complexity and diverse operating conditions of LoRaWAN, it is essential to gain insight into its actual internal functionality in real deployments using tools for network communication analysis. To address this need, we developed a dedicated hardware sniffer—a specialized device designed to capture and decode wireless traffic. In the context of LoRaWAN, this sniffer can be used to collect a dataset and subsequently investigate various aspects of the network, such as signal strength, coverage, data rates, and communication protocols. These insights can help identify potential issues, evaluate network deployments, and optimize configurations for better performance. To provide the greatest flexibility in analyzing the recorded packets, we selected Wireshark—a widely recognized open-source network protocol analyzer.

Our research is guided by several key questions related to the dataset. Firstly, we aim to determine which information can be extracted from captured real-world traffic within a LoRaWAN network, with particular attention to downlink traffic and Class-B beacons. Furthermore, we investigate how Class-B beacons and their optional extensions are used in actual installations. It is also crucial to assess whether security and spectrum regulations are followed in current LoRaWAN deployments. Another key aspect of our research is to examine the accuracy and reliability of time synchronization in LoRaWAN, notably regarding the Class-B beacons, and their susceptibility to interference and misconfiguration. Finally, we explore the potential for new applications of Class-B beacons.

### Contribution of This Work

We collected and analyzed a large dataset [6] of real-world LoRaWAN traffic from four European locations. Unlike previous datasets [7,8,9,10], our collection includes uplink, downlink, and Class-B traffic. In the Results and Discussion section, we present an analysis that encompasses the Class-B beacons and highlights potential issues of LoRaWAN deployments.

To obtain this dataset, we used a custom LoRaWAN hardware sniffer. Both the hardware and software sources of this device are available online [11]. Recognizing the outdated LoRaWAN protocol support in Wireshark, we enhanced its capabilities for decoding real-world traffic. These improvements are incorporated into the Wireshark development branch and are now publicly accessible.

Furthermore, we proposed an innovative approach of using Class-B beacons as a timebase source in urban environments. This method offers several advantages over alternative time sources such as Global Navigation Satellite System (GNSS), DCF77, and Network Time Protocol (NTP), including better indoor reception, smaller and more cost-effective antennas, and independence from internet connectivity.

Hence, the main contributions of this work are as follows:

- it describes a novel LoRaWAN sniffer with open hardware design files and software framework that allows capturing all LoRaWAN traffic and its examination in Wireshark;
- it provides a large public dataset with real-world traffic captured in multiple locations;
- it analyzes the unencrypted part of captured packets, providing insights into network operators, end device manufacturers, and LoRaWAN feature support;
- it provides an analysis of Class-B beacons regarding precise timing and gateway localization;
- it points out to several identified issues, like invalid Class-B beacons, compromised encryption keys, and invalid LoRaWAN traffic;
- it proposes the novel use of Class-B beacons as a timebase source.

## 2. Related Research

The IoT research community recognizes the significance of real-world, quantitative data for studying the network environments and deployments. Several LoRaWAN datasets have been made available [7,8,9,10]. Bhatia et al. [7] gathered uplink packets from gateways in the dense urban environment of London (UK). They included packet header information and PHY layer properties reported by the gateways, making the dataset one of the largest and most extensive [12]. Aernouts et al. [8] collected data focused on fingerprint localization in Antwerp (Belgium). Their dataset contains a large volume of traces with known end device position.

Blenn et al. [9] presented an analysis of The Things Network (TTN), obtaining a dataset of packets through the TTN Application Programming Interface (API) using a known default network key. However, their findings were constrained to TTN uplink traffic due to its API limitations. Presently, the acquisition of such dataset is no longer feasible due to the evolution of the TTN backend.

Choi et al. [10] developed LoRadar, a passive packet monitoring tool, and conducted an analysis of traffic within an anonymized city-wide area. Their study closely resembles our research. However, they were limited to monitoring uplink sessions due to hardware constraints. To the best of our knowledge, no previous study has attempted to capture both uplink and downlink simultaneously.

An overview of the existing sniffers is provided in [13]. These sniffers are limited to a single RF channel [14] or employ one multichannel concentrator [9,10]. They are either based on a gateway (concentrator type) [9,10] or developed using the GNU radio (SDR-type) [15]. Software-Defined Radios (SDRs) were deemed unsuitable due to high Signal-to-Noise Ratio (SNR) requirements [15,16,17]. Recently, an SDR-based demodulator competitive in SNR requirements was made available [18]. However, it still demodulates one frequency and Spreading Factor (SF) per block, requiring over 100 differently configured LoRa demodulator blocks for the intended sniffer functionality, which is computationally demanding.

Other papers focus on simulating various LoRaWAN issues (overview in [19]) and the deployment of custom experimental setups (controlled environments of nodes and one or multiple gateways) [20,21]. Our work focuses on passive monitoring of real-world traffic, similar to [10], but also includes an important study of downlink messages and Class-B beacons.

Time synchronization in LoRaWAN has been analyzed in several studies, such as [22,23]. Ramirez et al. [22] achieved an excellent time error below 10 μs using a custom protocol in a Class-A network. Rizzi et al. [23] employed a posteriori synchronization, enabling time sync with an uncertainty in the order of tens of milliseconds. No studies have suggested passive listening to Class-B beacons for time synchronization.

## 3. Sniffer Design

The sniffer is based on commercially available modules, and its software is customized for capturing network traffic. It operates autonomously when connected to a power source, storing the collected records locally and simultaneously transmitting them to a server over the Long Term Evolution (LTE) modem.

To overcome the limitations of currently available devices, our new sniffer needs to capture all LoRaWAN traffic according to the EU868 frequency plan, including the RX2 channel [24]. This necessitates supporting both uplink and downlink reception, which differ in the chirp signal polarity at the physical LoRa layer. Additionally, we aimed to receive Class-B beacons transmitted on RX2 channel with a non-inverted chirp signal. The combinations of these parameters are summarized in Table 1.

The sniffer is based on the industry-standard IMST iC880A LoRaWAN concentrator [27], a hardware device designed for receiving and processing LoRa signals in LoRaWAN network gateways. The module is equipped with a Semtech SX1301 digital baseband chip [28] and two Semtech SX1257 RF front end chips [29], providing up to 10 programmable parallel demodulation paths. It supports multiple LoRaWAN channels in the 868 MHz frequency band, enabling the simultaneous reception of data from multiple end devices. Additionally, the module is also capable of performing time-stamping of incoming packets, which is essential for precise time synchronization.

The main baseband chip SX1301 provides eight LoRa demodulators with automatic SF selection on IF0–IF7 signal paths. Moreover, an additional LoRa demodulator with fixed parameters and implicit header mode support, referenced as a SingleSF modem, is available on the IF8 signal path.

There are several limitations introduced by the chipset. In the LoRa physical layer, the modulated signal is represented by a chirp, which is a sinusoidal waveform whose frequency increases or decreases linearly over time [4]. The SX1301 demodulator can only detect chirps with one of two different polarities, each representing its inverse. Each LoRa demodulator needs to know the polarity of a LoRa chirp signal in advance. As a result, at least two iC880A concentrator modules need to be used for a simultaneous reception of uplink and downlink transmission, each configured to demodulate a different chirp signal polarity (GW #1 and GW #2).

**Figure 1:**
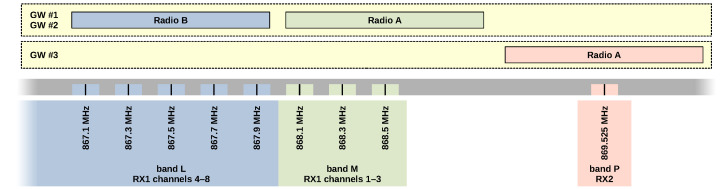
LoRaWAN EU868 channels and sniffer front ends.

The IF0–IF7 LoRa channels may be connected individually to radio front ends, referenced as Radio A or Radio B [27,28]. However, the useful bandwidth of SX1257 radios is approximately only 925 kHz [30], assuming typical 125 kHz channels in the EU868 band [24]. This bandwidth is sufficient for the simultaneous reception of all RX1 channels using both front ends. Nevertheless, the RX2 channel operates at a significantly different frequency, making it impossible to receive using the typical configuration. This is not an issue for a standard concentrator, as it only transmits on RX2 without receiving. However, for a sniffer, complete data reception is desired. To overcome this limitation, a third iC880A concentrator must be added to the sniffer system (GW #3). This concentrator enables reception in the RX2 downlink with one of its eight LoRa demodulators. Figure 1 illustrates the relationship between channels, bands, and radio front ends.

Another goal of the sniffer is to receive Class-B beacons. These beacons are transmitted on the RX2 frequency with specific parameters involving the implicit LoRa header [24]. Demodulation of the header is supported by the SingleSF modem on the IF8 signal path. An implicit header refers to a packet format where the length of the packet is not explicitly included in the packet header. Instead, the packet length is assumed to be fixed and known in advance. This reception is handled by the third concentrator module (GW #3).

### 3.1. Sniffer Hardware Overview

**Figure 2:**
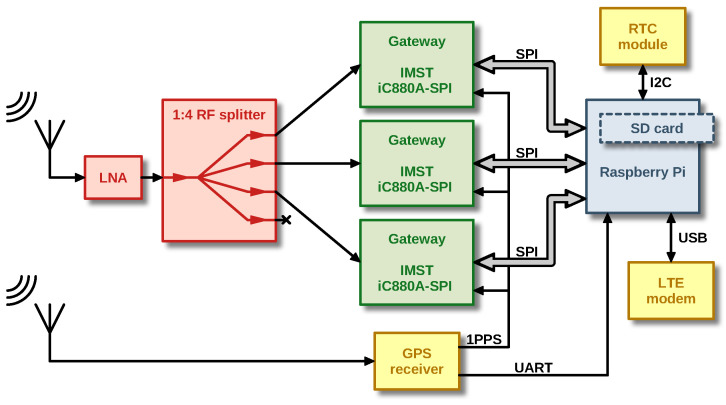
Block diagram of the developed LoRaWAN sniffer.

Figure 2 shows the block diagram of our LoRaWAN sniffer. Initially, the radio signal is received by an Ultra-High Frequency (UHF) omnidirectional antenna with a gain of 2 dBi and vertical polarization. This signal is subsequently filtered by a narrow bandpass filter, amplified by a Low Noise Amplifier (LNA), and then distributed to the inputs of three iC880A modules via a power splitter. Table 2 outlines the function of each iC880A module.

**Figure 3:**
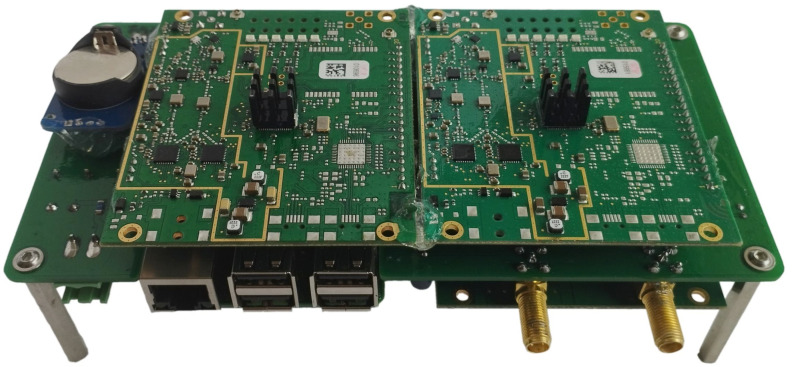
Photo of the LoRaWAN sniffer internal hardware.

A Raspberry Pi minicomputer serves as the central processing unit, which communicates with the iC880A modules through its integrated Serial Peripheral Interfaces (SPIs). In addition, it obtains the current time from a GNSS receiver module for accurate timestamping of the received packets. For this purpose, a 1 pps signal is distributed from the GNSS module to all iC880A concentrators. The Raspberry Pi also has a Real Time Circuit (RTC) connected to its I${}^{2}$C interface and an LTE modem connected via USB for remote management and sending the measured data to the server. An external 24 V adapter powers the whole device. Figure 3 shows the photo of the sniffer internal hardware. Complete schematics and hardware design files are available online [11].

From a mechanical perspective, the complete LoRaWAN sniffer is enclosed in an IP68-rated aluminum box, enabling safe outdoor installations. To accommodate the sniffer’s requirement for GNSS-based time synchronization and LTE communication, an additional plastic container conceals the GNSS and LTE antennas, eliminating the need for waterproof external antennas. The two containers are securely bonded together and all openings are sealed to maintain watertight integrity.

### 3.2. Sniffer Software Overview

The software relies on adapted open-source utilities supplied by Semtech, specifically libloragw from the lora_gateway repository [30] and lora_pkt_fwd from the packet_forwarder repository [31]. The LoRa gateway library manages SPI communication between the host computer and the SX1301 baseband chip. The packet forwarder employs the gateway library to receive packets, incorporate detailed data, and transmit the packet via a standardized UDP socket.

It was necessary to add support for handling multiple SPIs, switching chirp signal polarity, receiving packets without a valid Cyclic Redundancy Check (CRC), and decoding the Class-B beacon implicit header. As a result, the packet forwarder was modified to parse configuration JavaScript Object Notation (JSON) files and pass the relevant settings to the library, enhancing its versatility and adaptability. The complete software framework is available online [11].

### 3.3. Data Processing

To address the limitations of original Wireshark LoRa encapsulation, we developed an updated version of the LoRaTap header to efficiently manage the additional PHY layer information, such as frequency channel, signal level, timestamp, and other relevant details [32]. The sniffer’s JSON output produced by the packet forwarder utility can be converted to the pcap format by conversion utility [11].

We also significantly updated the Wireshark LoRaWAN dissector. Key enhancements include the addition of a LoRaWAN Class-B beacon dissector, *Join Accept* decryption, support for MAC commands from the LoRaWAN v1.0.4 specification [5], and various improvements to enable successful decoding of real-world traffic captured by the sniffer. These modifications are integrated into the development branch for future official release and are currently available through the Wireshark automated builds [33].

### 3.4. Analysis and Decryption

Subsequent data processing can be performed manually in Wireshark or through automated scripts in Wireshark’s console version, TShark. We employed an automated approach for the quantitative analysis of captured packets. Data post-processing from the TShark utility is executed with Python scripts, while final statistical and visual processing is carried out in MATLAB. The scripts are available online [11].

LoRaWAN packets are usually partially encrypted, with the keys generally unknown to a sniffer device. However, there are several properties of LoRaWAN communication that can be analyzed without knowing the decryption keys. The following fields of a LoRaWAN packet are not encrypted:

- Message Header (MHDR): Contains information about the message type (MType) and LoRaWAN version.
- Device Address (DevAddr): A unique 32-bit identifier for the end device within a specific network.
- Frame Control (FCtrl): Contains information about the Adaptive Data Rate (ADR), Frame Options Length, and other control flags.
- Frame Counter (FCnt): A 16-bit counter value that increments with each uplink frame to prevent replay attacks.
- Frame Options (FOpts): Contains optional MAC commands.
- Frame Port (FPort): Indicates the port number for application-specific or MAC layer communication.

The application payload (FRMPayload) and Message Integrity Check (MIC) are encrypted for both uplink and downlink packets, requiring the corresponding keys for decryption and verification [5].

LoRaWAN activation processes include the Over-the-Air Activation (OTAA) and Activation By Personalization (ABP). OTAA involves an end device transmitting a *Join Request*, encrypted with a pre-shared Application Key (AppKey). The network server verifies the request, generates session keys, namely the Network Session Key (NwkSKey) for the MIC and the Application Session Key (AppSKey) for the payload, and responds with a *Join Accept* message, which includes the assigned Device Address (DevAddr). Given the necessary keys, Wireshark can decrypt the join process packets, allowing for a more comprehensive analysis.

ABP, on the other hand, involves pre-configuring the end device with session keys (NwkSKey and AppSKey) and a DevAddr, enabling immediate communication without a join procedure. While this approach simplifies the process, it may increase security risks due to prolonged use of the same keys.

## 4. Results and Discussion

Data from the LoRaWAN networks were collected in four cities: Liege (Belgium), Graz (Austria), Vienna (Austria), and Brno (Czechia). These cities were chosen for data gathering due to various factors, such as their central European location, their prominence as major urban areas with well-established LoRaWAN networks, and the intention to capture a diverse range of city environments for data collection. Table 3 provides a summary of the characteristics and details associated with each capture.

**Figure 4:**
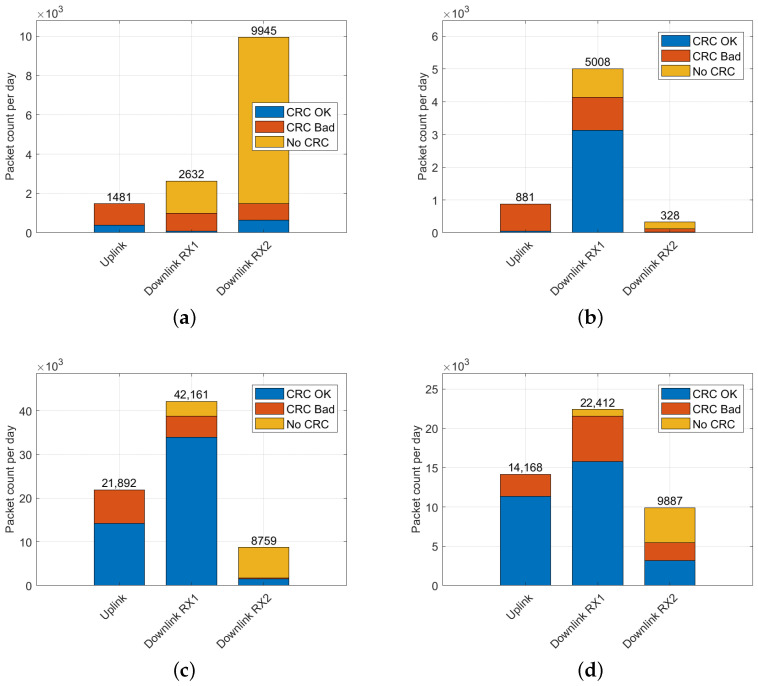
Distribution of LoRaWAN packets for individual receive chains for packets with valid, invalid, and missing CRC in: (**a**) Liege dataset; (**b**) Graz dataset; (**c**) Vienna dataset; (**d**) Brno dataset.

Ideal placement of the sniffer in Vienna and Brno is evident in the distribution of the number of packets received in uplink, downlink, and independent downlink (RX2), as depicted in Figure 4. To account for varying time periods across the datasets, packet counts in all histograms were normalized to display the number of packets per day.

In Liege, the site is primarily characterized by the downlink traffic—unconfirmed data without a checksum, particularly on the RX2 channel. The Graz data also suggest a suboptimal sniffer placement, as the sniffer predominantly captured downlink signals from gateways (better positioned than nodes). Consequently, most of the received uplink traffic was discarded due to wrong checksums, as shown in Figure 4.

### 4.1. Selected Results of Data Post-Processing

**Figure 5:**
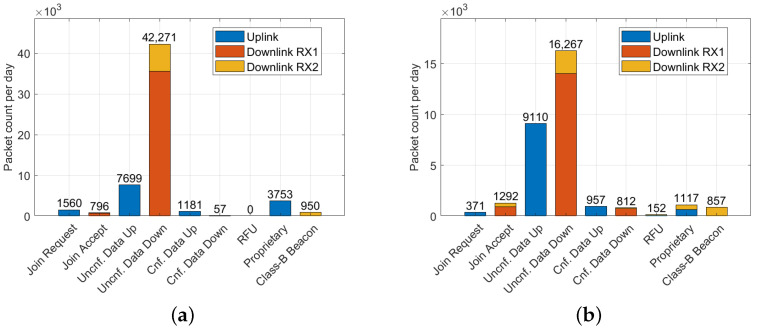
LoRaWAN message types: Join Request, Join Accept, Unconfirmed/Confirmed Data Up/Down, RFU, Proprietary, and Class-B Beacon in: (**a**) Vienna dataset; (**b**) Brno dataset.

Despite optimal sniffer placement in Vienna and Brno, a higher number of packets was received in the downlink compared to the uplink. The distribution of valid LoRaWAN message types is depicted in Figure 5. To determine the validity of real LoRaWAN messages, the CRC verification was applied at the physical LoRa packet level, and packet headers were checked for errors. Payload checksums were verified for the Class-B beacons.

Suboptimal placement in Liege and Graz resulted in the reception of predominantly downlink packets. Class-B beacons were observed in Brno, Liege, and Vienna. In some instances, particularly in Liege, these beacons also conveyed additional information regarding the geographic position of the gateway.

**Figure 6:**
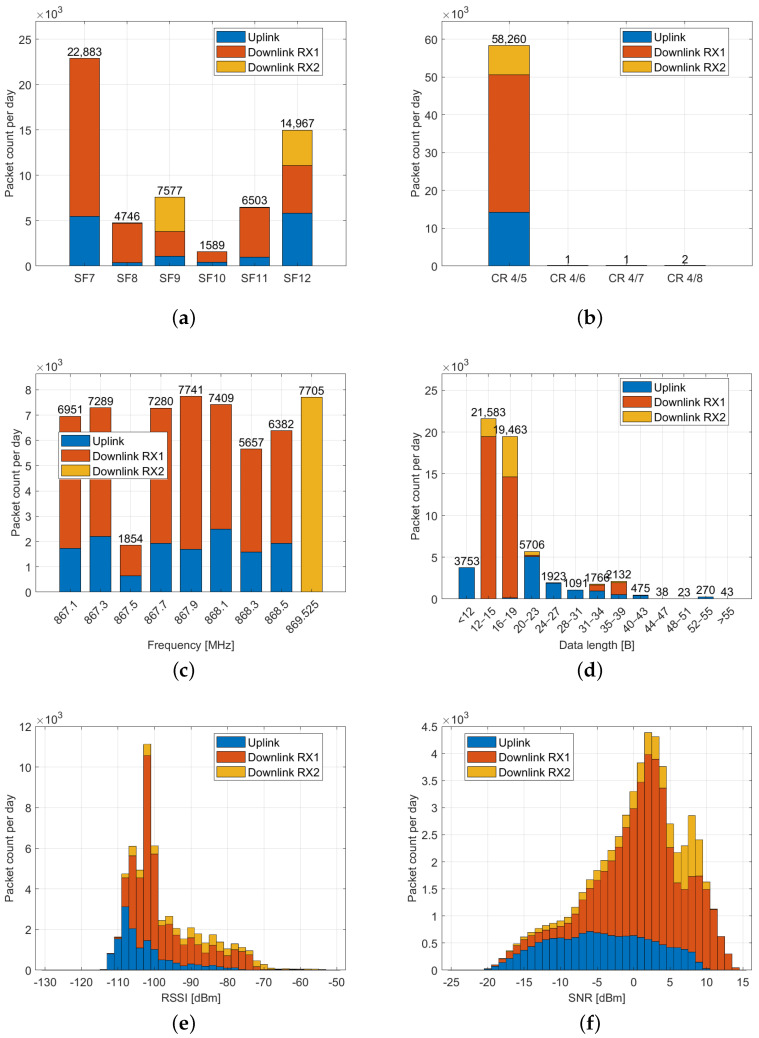
Parameters of captured LoRaWAN messages in the Vienna dataset: (**a**) Spreading factor; (**b**) Coding ratio; (**c**) Channel occupation; (**d**) Payload length; (**e**) Received Signal Strength Indicator (RSSI); (**f**) Signal-to-Noise Ratio (SNR).

The Vienna dataset can be considered a representative source of data. The histograms in Figure 6 demonstrate the identified transmission parameters. Spreading factors SF7 and SF12 are dominant, with a coding rate of 4/5 required by the standard [24]. Channels are occupied almost uniformly (except for the 867.5 MHz frequency), and most packets are relatively short, with lengths of 12–19 bytes in the downlink and 20–40 bytes in the uplink. The Received Signal Strength Indicator (RSSI) and the SNR confirm the superior placement of gateways compared to nodes in terms of radio coverage.

Table 4 shows the percentage of traffic with declared Adaptive Data Rate (ADR) support from end devices (extracted from uplink frames) and network servers (from downlink frames), declared end device Class-B support, and the percentage of downlink messages containing valid payload CRC.

ADR is a feature that optimizes the data rate, transmission power, and airtime for end devices based on their connectivity conditions [5]. In uplink frames, the ADR flag set by the end device indicates its support for the ADR feature and requests the network server to manage its data rate and transmission power settings. When the ADR bit is set in a downlink frame, it informs the end device that the network server can send ADR commands. The ClassB flag in the uplink packet header indicates to the network server that the end device activated Class-B mode and is ready to receive scheduled downlink pings.

In accordance with the LoRaWAN standard, uplink and downlink packets are distinguished by the presence of payload CRC. While payload CRC is mandatory in the uplink packets, the standard does not require it in the downlink, allowing for reduced airtime and associated duty cycle for gateway transmissions [5]. However, the observed data indicate that, aside from the Liege site, payload CRC is appended in the downlink by the majority of LoRaWAN gateways.

### 4.2. Network Operators

The DevAddr field serves as an identifier for the end device within the LoRaWAN network [5,34]. It is transmitted unencrypted in both the uplink and downlink. To determine the network operator, we cross-referenced the prefix of DevAddr with the LoRa Alliance list [35]. The corresponding findings are presented in Table 5, which highlights significant traffic (over 400 packets per day) from various locations. Identifying the source gateway from a captured downlink packet is infeasible without access to the network server because it lacks explicit gateway-related information.

### 4.3. End Device Manufacturers

In addition to analyzing the network operators, we also examined the end device manufacturers by analyzing the Device Extended Unique Identifier (DevEUI) field in the *Join Request* messages. The DevEUI is a globally unique number assigned to a LoRaWAN device and complies with the 64-bit Extended Unique Identifier (EUI-64) format [5].

To identify the manufacturers of end devices within the captured dataset, we cross-referenced the DevEUIs with the IEEE EUI-64 address space [36]. Table 6 presents the results of this analysis, highlighting the major traffic (over 10 join packets per day) from different manufacturers.

### 4.4. Class-B Beacon Analysis

In a LoRaWAN Class-B network, gateways must be synchronized to broadcast the Class-B beacons. Two possible modes of the transmission exist: tightly synchronized, with gateways synchronized to Global Positioning System (GPS) time with an accuracy better than 1 μs, allowing them to transmit beacons every 128 seconds; and loosely synchronized, where gateways can synchronize with GPS time with an accuracy better than 1 ms but not 1 μs, requiring randomized beacon transmission. Tightly synchronized gateways capitalize on the single-frequency network, while loosely synchronized gateways utilize randomization to counteract beacon interference caused by lower transmit timing accuracy.

To effectively filter the sniffer data, we utilized specific settings for beacon reception at the LoRa physical layer. These settings include an implicit header mode, SF9BW125, CR 4/5, no CRC, a payload length of 17 bytes, a preamble length of 10 symbols, and a non-inverted LoRa signal [24]. The payload comprises a timestamp (representing seconds elapsed since the start of the GPS epoch) and a gateway-specific parameter (e.g., geographic coordinates or network/gateway identification). Each part is protected by an independent CRC checksum.

Significant differences were observed between the various locations included in the dataset. Ideally, 675 beacons per day should be received, considering the beacon interval of 128 s and tightly synchronized gateways without transmitting randomization. As expected due to interference, the actual numbers were lower. However, the data from Vienna and Brno also contained a substantial number of packets violating the LoRaWAN standard, as discussed in later sections.

Table 7 presents an analysis of the captured LoRaWAN Class-B beacons across different locations. All packets included in the table have both of their CRC checksums valid. The timestamp correctness was determined by comparing the precise time of the beacon arrival and the timestamp value contained in its payload. No Class-B beacons were included in the captured data from Graz.

**Figure 7:**
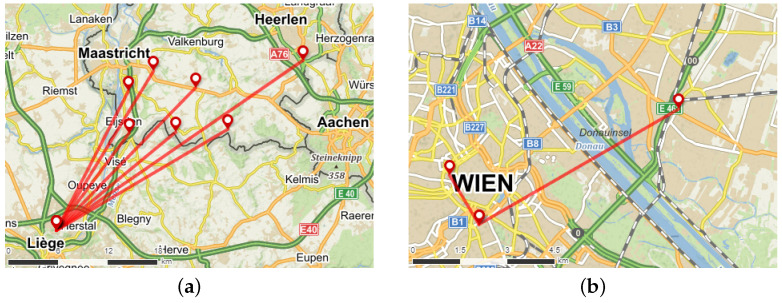
LoRaWAN sniffer placement and identified Class-B gateways beaconing its position in: (**a**) Liege; (**b**) Vienna. Map source: “Mapy.cz”.

The locations of Class-B gateways broadcasting their coordinates in the Liege and Vienna datasets, as well as lines connecting each gateway to the respective receiving sniffer, are depicted in Figure 7.

**Figure 8:**
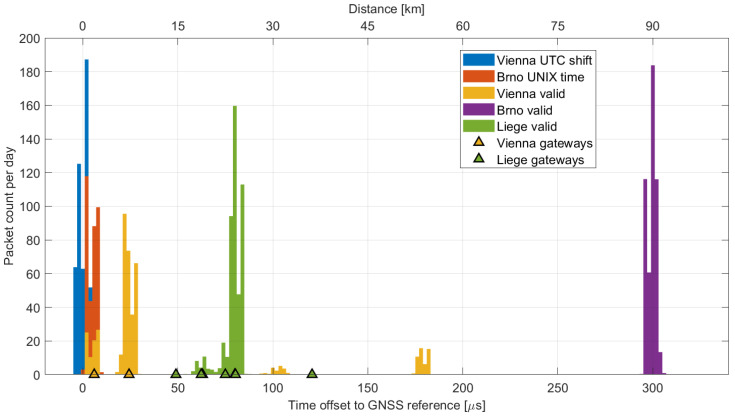
Time offset and corresponding distance between the GNSS reference and the received Class-B beacons.

One of the most crucial pieces of data obtained was the accuracy of the beacon timestamps. Figure 8 displays the difference between the sniffer reference time, synchronized by the 1 pps signal from the GNSS receiver, and the time of the received beacon packet. This difference should ideally represent the beacon signal propagation delay. The time of the received packet is adjusted by 154,143 μs to incorporate the following corrections:

- 1500 μs, the time delay specified in the LoRaWAN standard as $T_{B}eaconDelay$ [5];
- 152,576 μs, the beacon packet transmission time (calculated from the EU868 beacon channel settings [24] with tool [37]);
- 67 μs, the empirically determined delay, likely due to sniffer signal processing.

For gateways that broadcast their geographic position as part of the beacon payload, we calculated the distance from the sniffer and displayed it as triangular marks in Figure 8. The figure reveals the low jitter in the arrival times of beacons across all sites, enabling the identification of individual gateways based on their distance from the sniffer. In addition to the gateways confirmed through the geographic coordinates, Figure 8 displays the reception of two more gateways in Vienna (at distances of approximately 31 km and 58 km) and a gateway in Brno (at a distance of approximately 90 km). It is important to note that this distance calculation assumes tightly synchronized gateways. Gateways that appear to be significantly distant may be loosely synchronized, transmitting their beacons with a small delay.

It is also evident that the beacons with invalid timestamps described earlier are in close proximity to the sniffer. Considering the sniffer’s location on university campuses in both Vienna and Brno, these gateways might be experimental and utilized for research and development purposes.

#### 4.4.1. Beacons in the Liege Region

At the Liege location, well-configured gateways were found near the border in the Netherlands, specifically in the Maastricht and Haarlen area. A total of seven gateways with unique coordinates were identified, situated between 15 and 36 km from the sniffer. No gateways lacking the position or with invalid beacon frames were detected.

#### 4.4.2. Beacons in the Vienna Region

Two nearby gateways broadcasting their geographic coordinates were identified. Based on the timing analysis shown in Figure 8, it appears that two additional, more distant gateways also contributed to beacon broadcasting.

However, alongside valid packets, a number of frames transmitted at incorrect times were captured. The vast majority of these erroneous frames were offset by 18 seconds. This offset is likely due to a faulty implementation of the conversion between the GPS time used in Class-B beacons and the commonly used Coordinated Universal Time (UTC). GPS time does not include leap seconds [38,39] and was synchronized to UTC on 5 January 1980. In 2023, the number of leap seconds, i.e., the difference between GPS time and UTC, is precisely 18 s.

**Figure 9:**
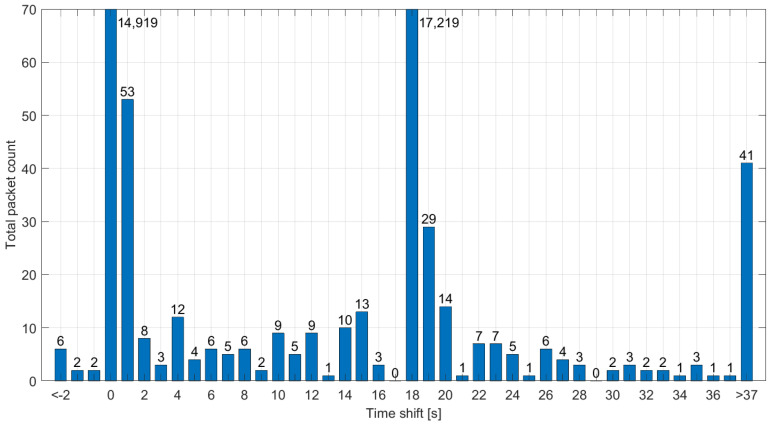
Difference between the actual reception time and the reported time in invalid Class-B beacons from the Vienna dataset.

Although the majority of recorded beacons were either valid or shifted by 18 s, the sniffer also recorded a significant number (0.9%) of packets with different time shifts, as demonstrated in Figure 9. The frames are always shifted by a whole number of seconds, meaning their synchronization within a one-second window is maintained. These packets are likely sent by a malfunctioning gateway, where the system clock is not correct, even though the actual beacon transmission is initiated accurately by the 1 pps signal. Together with the packets shifted by 18 seconds, they pose a significant issue for Class-B synchronization in the Vienna area and are likely to cause random network problems, resulting in LoRaWAN downlink latency degraded to Class-A. In this situation, the Class-B functionality of the end device depends on whether it synchronizes to a correct or invalid signal during the beacon acquisition phase [40].

#### 4.4.3. Beacons in the Brno Region

Two beacon signals were identified in the captured data from Brno. One correct signal was received from a gateway with a time offset corresponding to an approximate distance of 90 km from the sniffer’s location. No geographic coordinates that could confirm this distance were found, and approximately 0.1% of the frames were invalid.

Another beacon was identified at a distance of about 2 km. The timing information contained in this beacon was incorrect, shifted by 315,964,782 seconds. This value corresponds to the 315,964,800 seconds difference between the GPS and UNIX time. By subtracting the 18 leap seconds from this difference, we obtain the observed time shift. In other words, the gateway transmits a UNIX timestamp instead of the GPS time required by the LoRaWAN standard.

### 4.5. Channel Occupation and Duty Cycle Violations

LoRaWAN transmissions in the EU868 band must adhere to regulations specified in the ETSI EN 300 220 standard [25]. The duty cycle limitations for end devices and gateways vary depending on the specific frequency sub-bands. However, it should be noted that these duty cycle limitations are only required if the Listen Before Talk (LBT) is not used:

- band L, 865 MHz to 868 MHz, ≤1% duty cycle, includes RX1 channels 4 to 8;
- band M, 868.000 MHz to 868.600 MHz, ≤1% duty cycle, includes RX1 channels 1 to 3;
- band P, 869.400 MHz to 869.650 MHz, ≤10% duty cycle, includes RX2 channel.

The EU868 band is shared with other short-range devices that comply with the regulations, typically employing narrow-band Frequency Shift Keying (FSK) and Amplitude Shift Keying (ASK) modulations. We assessed the shared spectrum usage by calculating the on-air time of each captured packet, a value derived from the spreading factor, bandwidth, coding rate, preamble length, and packet length [37,41].

**Figure 10:**
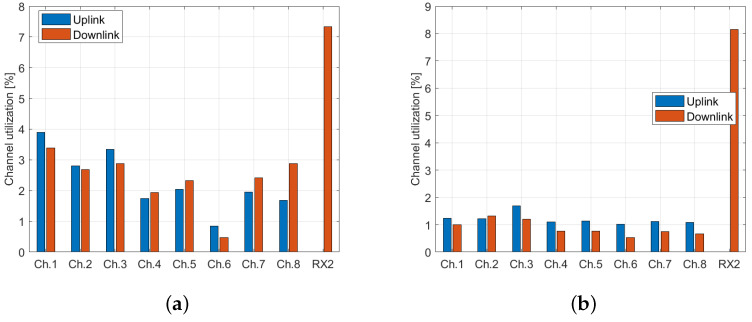
Channel utilization by LoRaWAN packets in (**a**) Vienna dataset; (**b**) Brno dataset.

Furthermore, we computed the total air time for all sites, separately for the uplink and downlink, since different transmit directions utilize inverted, uncorrelated chirps. All captured packets, including those with invalid CRCs, were considered in the calculation to evaluate the total LoRa transmission time on the respective channel. The highest channel occupation was observed at Vienna and Brno, as shown in Figure 10.

Associating a downlink packet with a specific gateway is unfortunately not feasible. Nonetheless, it is possible to calculate the uplink on-air time for individual end devices based on their DevAddr field. The ETSI EN 300 220 duty cycle limits are not applicable to individual LoRaWAN channels; instead, they apply to specified bands that encompass several adjacent channels.

Considering this criterion, we identified a total of eight devices in the Brno dataset that, while adhering to the 1% duty cycle limitation on individual channels, exceed this limitation by several times for the L and M bands (with duty cycles reaching up to 3.9% for the L band and up to 2.6% for the M band). In the Vienna dataset, a single device was discovered to violate the limitation (2.7% duty cycle in the L band). However, it is worth noting that the devices we identified as exceeding the duty cycle limitation could potentially be using the LBT strategy.

### 4.6. Compromised Encryption Keys

The captured data show that exposed encryption keys are used in existing LoRaWAN networks. Semtech’s default key (2B7E151628AED2A6ABF7158809CF4F3C) [9] was identified in the Brno dataset. This key is used as the AppKey for the OTAA by RisingHF devices by default, according to the DevEUI identifier. A significant number of data packets (15.5% of all valid packets) from ABP-activated devices in Brno use it as both the NwkSKey and AppSKey. A smaller number of such devices were also discovered in the Vienna dataset (0.2%).

Similarly, the Milesight default key (5572404C696E6B4C6F52613230313823) [42] was identified in OTAA-activated devices across Vienna, Brno, and Liege, primarily utilizing TTN. If an eavesdropper intercepts the entire *Join Request*–*Join Accept* pair, they could derive the NwkSKey and AppSKey, enabling them to decrypt the entire communication of the affected device.

A small number of packets in the Brno and Vienna datasets were also found to use the empty key (00000000000000000000000000000000). In these cases, the devices appear to be unconfigured or experimental.

### 4.7. Limited Front End Image Frequency Rejection

**Figure 11:**
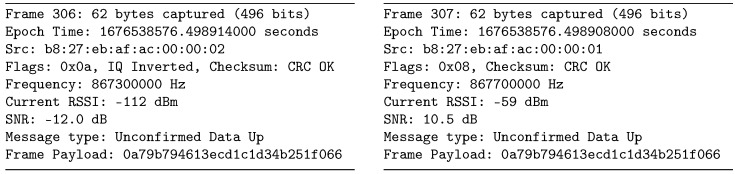
Duplicate packets with different chirp polarities in the Brno dataset.

Strong packets can sometimes be received on two different channels with different chirp polarities. This phenomenon arises due to the limited value of the Image Frequency Rejection Ratio (IMRR) of the radio front ends found in LoRaWAN gateways and the sniffer. An example of this can be identified in packets #306 and #307 in the Brno dataset, with the key characteristics depicted in Figure 11.

The packets were received almost simultaneously, with a negligible 6 μs difference. Frame #307 is a valid uplink transmission with a signal strength of −59 dBm and a non-inverted chirp. Given a received frequency of 867.7 MHz and a front end center frequency of 867.5 MHz (as shown in Table 1), we can anticipate a mirror signal at 867.3 MHz. This is confirmed by frame #306, which has a signal strength of −112 dBm. The difference of 53 dB corresponds to the IMRR value of the SX1257 front end employed in the sniffer.

Due to the signal spectrum inversion, such invalid packets can be easily identified by the inverted chirp flag, because data marked as uplink in the LoRaWAN header should not be detected by the downlink sniffer. To ensure data accuracy, these packets were filtered out during processing. The described behavior could potentially overshadow a legitimate weak packet at the mirror frequency. However, the likelihood of its occurrence is almost negligible, given the low usage of the channels.

### 4.8. Invalid LoRaWAN Traffic with Valid Checksum

LoRa packets at the PHY layer contain a Synchronization Word (SyncWord), which serves to differentiate the contents of the following payload. The use of the SyncWord can be confusing due to limited information from the manufacturer.

Semtech recommends only two SyncWord values: 0x12 for private networks, and 0x34 for public/LoRaWAN networks [41,43]. Documents from the LoRaWAN Alliance [24] and certain source codes [30] imply that SyncWord 0x34 is designated for all networks utilizing the LoRaWAN protocol at the MAC layer. This interpretation suggests that both publicly and privately designed networks following the LoRaWAN standard should employ SyncWord 0x34. The private SyncWord 0x12 appears to be reserved for devices utilizing LoRa modulation at the PHY layer without engaging the LoRaWAN MAC layer.

The SyncWord setting is crucial for both modulation and demodulation, as the receiver does not accept packets transmitted with a different SyncWord [44]. This issue is not merely about discarding packets in the case of a mismatch; it arises from the inability to synchronize on the preamble–SyncWord pair [45].

All datasets contain packets with errors that the LoRaWAN dissector cannot decode. The Liege dataset includes a significant number of invalid packets (4.7% of the total). Invalid packets are identified by dissector errors or invalid MAC header entries, which include a non-zero Reserved for Future Use (RFU) field and a Major version that is not equal to R1.

Receiving an invalid LoRaWAN packet can be attributed to a misconfiguration of the LoRa transmitter, which uses a custom payload for packets set with a public SyncWord. The correct approach would be to use a dedicated private SyncWord, which appears to be the issue occurring in the Liege dataset. Another possibility involves accepting invalid packets that are erroneously evaluated as valid due to various factors. This could be attributed to the limited reliability of the 16-bit payload CRC [41], which may occasionally fail to identify packet corruption, or it could be due to the unwanted acceptance of packets with a private SyncWord.

The SyncWord issue was investigated in the InterOP project ATCZ175 [46], and its results indicate a relatively low capability of the gateway to filter packets based on SyncWord. The success rate of receiving a private SyncWord packet when the gateway is set to the public SyncWord depends on the signal strength and the SF used, with the possibility of reaching up to 10%. Consequently, any traffic with a private SyncWord may lead to the observation of invalid packets in sniffer datasets.

### 4.9. Class-B Beacons as a Timebase Source

Class-B beacons in LoRaWAN networks have the potential to serve as alternative timebase source in urban environments. Beacon receivers typically lock within 128 s, providing excellent long-term stability, as their timing is usually derived from a GNSS receiver. According to the LoRaWAN standard, beacon timing is accurate within ±1 μs, while measurements taken by the sniffer without further optimizations revealed an accuracy of ±5 μs. This accuracy is further reduced by the wireless propagation delay—every 300 m of distance represents an additional 1 μs offset.

Compared to GNSS, Class-B beacons can be received indoors, making them suitable for time synchronization in buildings and other structures where GNSS signals are weak or unavailable. Unlike GNSS, Class-B beacons do not require a clear view to the sky, enhancing their reliability in urban environments where tall buildings, trees, or other obstacles might obstruct GNSS signals.

Class-B beacons also offer several benefits compared to the DCF77, a Long-Wave (LW) time signal broadcast from Germany. They exhibit high immunity to noise, making them more reliable in urban environments where specific types of RF interference are common, e.g., the LW interference affecting DCF77 signals. Class-B beacon receivers can use small, cheap antennas, lowering the overall cost and making them more accessible for a wide range of applications. Moreover, Class-B beacons have a similar lock speed to DCF77, with a lock time of up to 128 s compared to DCF77’s typical lock time of 2–3 min [47].

Compared to NTP, Class-B beacons do not require an internet connection for time synchronization, making them suitable for environments with limited or no internet access. This independence from internet connections makes Class-B beacons a compelling alternative for various applications.

To further enhance the lock time, a multichannel (e.g., SDR-based) device may listen for Class-A downlink traffic, which may contain the *DeviceTimeAns* time command in its unencrypted MAC header. Despite the limited accuracy of ±100 ms as defined in [5], this may allow for a coarse lock. The listening device can also derive the time window for Class-B beacon reception from this information, potentially reducing continuous receive time.

However, this proposed time synchronization may encounter difficulties if nearby gateways transmit beacons that violate the LoRaWAN standard. Such issues have already been observed in the Vienna and Brno regions, as previously discussed. Currently, no method exists to verify the authenticity of a received beacon. Moreover, due to the harsh RF environment, beacons may be disrupted by a wide-band UHF interference, resulting in decoding errors and significantly longer lock time.

Despite these challenges, by leveraging the benefits of Class-B beacons, time synchronization in urban environments can be significantly improved. The indoor reception capabilities, noise immunity, cost effectiveness, and independence from satellite availability and internet connections make Class-B beacons an attractive alternative for existing time synchronization methods, provided that the associated disadvantages can be effectively managed.

## 5. Conclusions

In this study, we created an extensive, publicly available dataset encompassing complete LoRaWAN traffic from four European cities. This dataset enabled rigorous examination of real-world LoRaWAN network functionality. Our analysis revealed security and system challenges, which include:

- invalid Class-B beacon packets, which pose a significant synchronization issue and are likely to cause random Class-B network problems;
- default encryption keys from Semtech and Milesight in existing LoRaWAN installations, which pose a security risk;
- end devices violating the duty cycle limitation for EU868 sub-bands, which could potentially degrade the quality of service for other wireless devices.

We enhanced Wireshark’s LoRaWAN protocol dissector to accurately decode recorded traffic, including data and MAC command decryption for packets with known keys. These improvements are now publicly accessible. Additionally, we proposed the use of Class-B beacons as a timebase source in urban environments.

Future research should incorporate datasets from a broader range of locations to enhance understanding of LoRaWAN networks. Additionally, addressing the issues related to invalid Class-B beacons is a critical next step. Class-B devices currently allow the fallback to Class-A when they experience difficulties in tracking the beacon. However, this depends on the specific device implementation, since the documentation only suggests an initial non-specific synchronization [5,40].

Validating received beacons remains a challenge. The beacon payload may contain an optional network/gateway identification. However, to the best of our knowledge, no beacon filtering implementation has been introduced yet. Another approach could involve transmitting the initial synchronization over a secure channel, specifically within a unicast packet with a MIC signature. This method can be employed to acquire the correct Class-B beacon. While a solution that utilizes the *DeviceTimeAns* command to acquire coarse time has been implemented, its use remains optional.

## Figures and Tables

**Table 1:** LoRaWAN EU868 frequency plan with possible combinations of LoRa parameters [24,25,26].

Transmission Kind	Frequency (MHz)	Spreading Factor	Uplink Signal Polarity	Downlink Signal Polarity
RX1 channel 1	868.5−0.4=868.1	SF7–SF12	non-inverted	inverted
RX1 channel 2	868.5−0.2=868.3	SF7–SF12	non-inverted	inverted
RX1 channel 3	868.5	SF7–SF12	non-inverted	inverted
RX1 channel 4	867.5−0.4=867.1	SF7–SF12	non-inverted	inverted
RX1 channel 5	867.5−0.2=867.3	SF7–SF12	non-inverted	inverted
RX1 channel 6	867.5	SF7–SF12	non-inverted	inverted
RX1 channel 7	867.5+0.2=867.7	SF7–SF12	non-inverted	inverted
RX1 channel 8	867.5+0.4=867.9	SF7–SF12	non-inverted	inverted
RX2	869.525	SF7–SF12 ^1^	–	inverted
Class-B beacon ^2^	869.525	SF9	–	non-inverted

^1^ SF12 for the LoRaWAN standard, SF9 for The Things Network [26]. The sniffer supports all spreading factors. ^2^ Class-B beacons use implicit header mode with specific settings [24].

**Table 2:** Roles of iC880A modules in the LoRaWAN sniffer.

Concentrator	Receives on	IF0–IF7 Paths	IF8 Path
GW #1	RX1 channel 1–8	downlink	–
GW #2	RX1 channel 1–8	uplink	–
GW #3	RX2	downlink (IF0 only)	Class-B beacon

**Table 3:** Dataset details.

Location	Geographic Coordinates	Sniffer Placement	Capture Interval	Days	Average Packets per Day	Valid LoRaWAN Packets per Day
Liege (Belgium)	50.66445° N 5.59276° E	Roof of a residential building in a suburb area; limited view.	25 August 2022–19 September 2022	17.8	14,088	6609
Graz (Austria)	47.07049° N 15.44506° E	Enclosed balcony of a historical building in the city center; indoor.	26 October 2022–29 November 2022	26.3	6225	3215
Vienna (Austria)	48.19666° N 16.37101° E	Roof of a university building in the city center; clear view.	1 December 2022–4 January 2023	34.1	72,892	58,330
Brno (Czechia)	49.22685° N 16.57536° E	Roof of a university building in a suburb area; clear view.	16 February 2023–30 March 2023	42.0	46,467	30,937

**Table 4:** Support for ADR and Class-B features along with the occurrence of payload CRC in downlink messages found in captured LoRaWAN messages.

Location	Gateway Packets with ADR Support	End Device Packets with ADR Support	End Device Packets with Class-B Support	Downlink Messages with Payload CRC
Liege (Belgium)	3.9%	79.8%	2.3%	1.2%
Graz (Austria)	99.7%	57.4%	34.1%	99.7%
Vienna (Austria)	79.2%	83.6%	1.4%	81.9%
Brno (Czechia)	96.6%	86.6%	0.0%	99.3%

**Table 5:** Major network operators identified from the captured LoRaWAN traffic.

Network Operator	Liege		Graz		Vienna		Brno	
	Packets per Day	% of Total	Packets per Day	% of Total	Packets per Day	% of Total	Packets per Day	% of Total
Private/experimental nodes	1312	21.6	3152	99.1	33,886	66.1	19,759	72.8
Minol ZENNER Connect	–	–	–	–	6138	12.0	–	–
The Things Network	59	1.0	9	0.3	4600	9.0	2194	8.1
Proximus	2751	45.3	–	–	4158	8.1	–	–
Actility	972	16.0	–	–	–	–	–	–
KPN	757	12.5	–	–	–	–	–	–
Orbiwise	–	–	–	–	412	0.8	–	–
Other/unassigned	218	3.6	19	0.6	2070	4.0	5193	19.1

**Table 6:** Major end device manufacturers identified from the captured LoRaWAN traffic.

End Device Manufacturer	Liege		Graz		Vienna		Brno	
	Packets per Day	% of Total	Packets per Day	% of Total	Packets per Day	% of Total	Packets per Day	% of Total
DZG Metering	–	–	–	–	603	38.6	–	–
RisingHF	–	–	–	–	–	–	288	77.7
Milesight	13	26.2	<1	0.7	106	6.8	7	1.9
Microchip Technology	–	–	–	–	93	6.0	6	1.6
Invoxia	1	1.9	–	–	90	5.8	–	–
Laird Connectivity	–	–	–	–	74	4.7	–	–
Adeunis RF	–	–	–	–	45	2.9	<1	∼0
MClimate	–	–	–	–	2	0.1	42	11.4
Holley Metering	–	–	–	–	30	1.9	–	–
ELSYS	–	–	–	–	23	1.5	–	–
Dragino Technology	–	–	–	–	17	1.1	<1	∼0
Seeed Technology	–	–	–	–	12	0.8	–	–
Viloc	11	21.8	–	–	–	–	–	–
Homerider Systems	10	18.8	5	43.0	1	0.1	–	–
Other/unassigned	16	31.4	9	56.3	893	29.9	65	7.4

**Table 7:** Analysis of the captured LoRaWAN Class-B beacons.

Timestamp	Liege		Graz		Vienna		Brno	
	Packets per Day	% of Total	Packets per Day	% of Total	Packets per Day	% of Total	Packets per Day	% of Total
Correct (includes location)	484	100.0	–	–	383	40.3	–	–
Correct (no location)	–	–	–	–	55	5.8	495	57.7
Incorrect, shifted by 18 s	–	–	–	–	505	53.1	–	–
Incorrect, in UNIX format	–	–	–	–	–	–	361	42.1
Incorrect (other)	–	–	–	–	9	0.9	1	0.1

## Data Availability

The data presented in this study are openly available on Zenodo at 10.5281/zenodo.8090619, reference number [6].
